# Flavivirus Zika NS4A protein forms large oligomers in liposomes and in mild detergent

**DOI:** 10.1038/s41598-024-63407-y

**Published:** 2024-05-31

**Authors:** Wahyu Surya, Shwe Sin Honey, Jaume Torres

**Affiliations:** School of Biological Sciences, Nanyang Technological University, 60 Nanyang Drive, Singapore, 637551 Singapore

**Keywords:** Flavivirus, Zika virus, Dengue virus, Non-structural protein 4A, Oligomerization, Analytical ultracentrifugation, Molecular conformation, Molecular modelling

## Abstract

In flaviviruses such as Dengue or Zika, non-structural (NS) NS4A protein forms homo-oligomers, participates in membrane remodelling and is critical for virulence. In both viruses, mature NS4A has the same length and three predicted hydrophobic domains. The oligomers formed by Dengue NS4A are reported to be small (*n* = 2, 3), based on denaturing SDS gels, but no high-resolution structure of a flavivirus NS4A protein is available, and the size of the oligomer in lipid membranes is not known. Herein we show that crosslinking Zika NS4A protein in lipid membranes results in oligomers at least up to hexamers. Further, sedimentation velocity shows that NS4A in mild detergent C14-betaine appears to be in fast equilibrium between at least two species, where one is smaller, and the other larger, than a trimer or a tetramer. Consistently, sedimentation equilibrium data was best fitted to a model involving an equilibrium between dimers (*n* = 2) and hexamers (*n* = 6). Overall, the large, at least hexameric, oligomers obtained herein in liposomes and in mild detergent are more likely to represent the forms of NS4A present in cell membranes.

## Introduction

Zika virus (ZIKV) was first isolated from the serum of a monkey in the Zika Forest of Uganda, almost 70 years ago^1^. Transmission to America resulted in more than a million people infected in 2015^2^. Since then, 89 countries have experienced outbreaks^3^. ZIKV is a single‐stranded positive‐sense RNA virus that belongs to the *Flavivirus* genus in the *Flaviviridae* family. This genus also includes Yellow fever virus (YFV), West Nile virus (WNV) and Dengue Fever virus (DENV)^4^. *Aedes* mosquitoes are the vectors for natural transmission cycle^5–7^ but human-to-human transmission has been also reported^8–10^.

About 80% of ZIKV-infected people are asymptomatic and others suffer influenza-like syndrome^11^, but thousands of cases have been reported of microcephaly and other neurological disorders^12–16^. Indeed, TLR3 activation leads to gene expression changes and neurogenesis disruption^17^, and ZIKV is known to reduce the viability of human brain cells^18^. Infection has been associated with Guillain–Barre Syndrome (GBS)^19^, an autoimmune disease that can be lethal^20^, although the link is not confirmed^21^. There are no vaccines nor antiviral agents available to treat ZIKV infections^22,23^. Therefore, the development of vaccines and antivirals against ZIKV is a current relevant challenge.

Flaviviruses encode a long polyprotein that is eventually cleaved by proteases into structural proteins: capsid (C), premembrane/membrane (prM/M) and envelope (E) and non-structural (NS) proteins NS1, NS2A, NS2B, NS3, NS4A, NS4B and NS5^24^. The detailed functions of most ZIKV proteins have not been studied but they are likely to be similar to those found in other flaviviruses such as DENV^25–27^. In members of this genus, RNA synthesis takes place in the replication complex (RC), a virus-induced membrane network derived from the endoplasmic reticulum (ER)^28–30^ that contains non-structural (NS) proteins, viral RNA and host cell factors^24,31,32^. After entering the cell^7^ ZIKV is internalized in endosomes. As in other flaviviruses, low pH triggers the fusion of viral envelope and endosomal membranes probably mediated by the E protein^33^. The C protein associates to genomic RNA to form the virion core, whereas prM assists E protein folding while preventing its premature fusion activity^34^. Virions bud into the ER to form enveloped immature virions that traffic through the Golgi complex^33,34^.

NS4A protein is sufficient to induce membrane alterations that resemble the highly curved membranes typical of RCs^32^. Like DENV NS4A, ZIKV NS4A consists of 127 residues, with residues 1–48 corresponding to a hydrophilic cytoplasmic domain, and three predicted TM segments according to TMHMM prediction^35^, encompassing residues 51–73, 78–97 and 102–121, respectively. Isolated N-terminal cytoplasmic domains of NS4A in DENV and ZIKV (residues 1–48) were reported to be disordered in solution, but become ~ 50% α-helical in presence of detergent or lipid membranes^36–39^. This N-terminal domain has been linked to cytopathic effects^40–42^.

The roles of NS4A in flaviviruses encompass membrane remodelling and other biochemical aspects that are critical during infection, as summarized recently^43^. Indeed, flavivirus infection causes a dramatic membrane rearrangement referred to as paracrystalline arrays, convoluted membranes or vesicle packets which are important during replication. For example, in DENV and WNV, NS4A protein expression is strictly required to induce membrane rearrangements^32,44^.

An intermediate of polyprotein processing is NS4A-2K-NS4B, where 2K is a transmembrane peptide subsequently cleaved by a viral protease, releasing mature NS4A^32^. This cleavage has been suggested to be important in the induction of membrane rearrangements^32,44^, and may be accompanied by a conformational change and/or oligomerization of NS4A.

Solution NMR studies have determined the α-helical regions of NS4A in DENV-4. The N-terminal tail contains three helices (α1–α3)^45^, followed by three α-helical hydrophobic domains (α4, α5 and α6)^45^ which correspond to the earlier predicted ‘putative transmembrane domains’ (pTM1–3). However, biochemical data is consistent with only pTM1 and pTM3 spanning the membrane, whereas pTM2 would be embedded, but not spanning the bilayer^32^. These authors proposed that membrane curvature could be generated by the wedge-like insertion of pTM2 into the luminal leaflet of the ER membrane, possibly triggered by NS4A oligomerization^32^. Consistently, a faster NMR hydrogen/deuterium exchange was observed in pTM2^45^ in detergents, suggesting that this helix is not membrane-spanning.

ZIKV NS4A protein has exactly the same length (127 residues) as DENV-4 NS4A, and similar predicted topology and TM regions, identity of 41.73% and similarity of 63.78%. In flaviviruses, NS4A protein forms at least homodimers on the basis of multiple bands observed in SDS gels^36,42,46–48^ and from pull-down assays^48^. Both the hydrophilic N-terminal domain^36^ and pTM1 (α4, residues ~ 50–76) have been suggested to be important for homo-oligomerization^48^. Since both domains have been mutated extensively and are important for replication and viral production^40,48,49^, disruption of these inter-molecular interactions constitutes a potential avenue for antiviral discovery^50–52^. However, neither the precise function nor the size of these NS4A homo-oligomers is clear. Various oligomeric forms have been reported in the literature^36,42,45,46,48^, usually dimers, but also higher. However, these observations have been performed in SDS, a harsh detergent that does not retain the native conformation of proteins and is therefore unsuitable to study oligomerization. In addition, membrane proteins can experience anomalous mobility in SDS and artefactual aggregates due to the detergent. Attempts to crosslink full length NS4A^45^ produced mostly monomers and dimers, although these were performed in detergent dodecylphosphocholine (DPC) at a very high detergent to protein ratio (DPR) of 400 which favours monomeric forms.

Therefore, herein we have tested the oligomerization behaviour of ZIKV NS4A protein using crosslinking of the protein in liposomes, and analytical ultracentrifugation (AUC) in micelles of C14-betaine, a mild detergent that has been successfully used previously in other transmembrane systems by us^53–55^ and others^56–58^. AUC measures protein–protein interactions in solution, does not require any calibration, and does not suffer from protein interaction with matrices. Disruption of the observed interactions may be used as an assay to test much needed antivirals.

## Methods

### ZIKV NS4A expression and purification

The full-length Zika NS4A protein (16.5 kDa, strain MR-766, accession YP_002790881.1), hereafter named NS4A, was expressed and purified with an N-terminal His-tag in decyl maltoside detergent (DM, Anatrace), as described previously^59^. Measurement of protein concentration was as previously described^59^.

### Crosslinking and SDS-PAGE

Crosslinking reagents disuccinimidyl glutarate (DSG), bis(sulfosuccinimidyl) suberate (BS3), disuccinimidyl suberate (DSS), ethylene glycol bis(succinimidyl succinate) (EGS), and dithiobis(succinimidyl propionate) (DSP) were obtained from Thermo Fisher Scientific (Singapore). Purified NS4A (12 µM) in detergent was mixed with a 4:1 molar ratio mixture of POPC/POPS (Avanti Polar Lipids) with final lipid-to-protein ratios (LPR) of 40, 100 and 250, followed by 5 min sonication. Crosslinkers at increasing concentrations (0.03 to 1.0 mM) were added to liposome samples followed by 15 min incubation. The reaction was terminated by addition of Tris pH 7.4 and LDS sample buffer (Thermo Scientific). Crosslinked samples were separated by SDS-PAGE in a 10% Bis–Tris gel with MOPS running buffer and transferred onto PVDF membrane (Pall Filtration, Singapore). Western blot detection was done using anti-His-tag (Thermo Scientific) antibodies and visualized by chemiluminescence with Pierce ECL Western Blotting Substrate (Thermo Scientific) using an Amersham ImageQuant 800 imaging system.

### Analytical ultracentrifugation (AUC)

AUC data were collected on a Beckman ProteomeLab XL-I Analytical Ultracentrifuge with an 8-place An-50 Ti analytical rotor. For both SV and ES experiments, samples were prepared in the same way, i.e., keeping the detergent concentration constant (1 mM) and changing the protein concentration, as follows: NS4A samples were first dialysed into 50 mM Tris–HCl pH 7.3, 100 mM NaCl, 2 mM TCEP (3-[bis(2-carboxyethyl)phosphanyl]propanoic acid;hydrochloride, Sigma), and 1 mM C14-betaine (*N*-Tetradecyl-*N,N*-dimethyl-3-ammonio-1-propane sulfonate or sulfobetaine-14, Sigma) for three days at 4 °C. Different detergent-to-protein ratios (DPR) were prepared by serial dilution of dialysed sample starting from DPR 20 (50 µM NS4A) to DPR 40, 80, 160, 320 and 640, in the same buffer containing 33.4% D_2_O to eliminate detergent buoyancy.

The matching D_2_O concentration used deserves a comment here. The matching D_2_O concentration depends on exact experimental conditions but it has been cited as 29.4% (v/v)^60^ and experimentally explicitly determined previously by us as 29.4%^61^ or 32.3%^62^. We note, however, that for C14-betaine in buffers similar to ours, publications from the labs of W.F. Degrado and K.G. Fleming have reported a matching D_2_O concentration as low as 13%^57,58,63–65^, without providing any explicit experimental determination. Thus, herein we again experimentally obtained the matching D_2_O concentration for C14-betaine in 50 mM Tris and 100 mM NaCl to be 33.4% (Supplementary Fig. S1A–C), close to the value our lab determined previously^62^. A matching D_2_O concentration of 13% has also been used for C14-betaine in 20 mM Tris and 200 mM KCl^66^, but we show here that in this case the matching D_2_O concentration should be also higher than 30% (Supplementary Fig. S1D). Therefore, we conclude that density-matching D_2_O for C14-betaine must be in the range 29–34% and not 13%.

For sedimentation velocity (SV) experiments, samples were loaded into AUC cells fitted with 2-sector Epon centerpiece and quartz windows. The cells were centrifuged at 50,000 rpm (25 °C) and absorbance scans at 230 nm (for more diluted samples with DPR 640, 320, and 160) or 280 nm (for samples with DPR 80, 40, and 20) were collected every 5 min for 10 h. We note that the detergent used herein, C14-betaine, does not absorb at 230 or 280 nm (Supplementary Fig. S2). SV data was analysed using *c(s)* size distribution models in SEDFIT^67^. S-values were normalized to standard condition s_20,w_ using buffer density, viscosity and protein partial specific volume calculated using SEDNTERP^68^.

### S-value range estimation

A range of possible s-values corresponding to the different NS4A-FL oligomers in C14-betaine micelles was estimated as described^69^ using the following Eq. (1)^70^:

1 $$s=\frac{{M}_{p }\left[(1-{\overline{\nu }}_{p}\rho + {\delta }_{d} (1-{\overline{\nu }}_{d}\rho )\right]}{{N}_{A}6 \pi \eta \left(\frac{{f}_{exp}}{{f}_{min}}\right) {\left[\frac{{3M}_{p }({\overline{\nu }}_{p}+{\delta }_{d} {\overline{\nu }}_{d})}{4\pi {N}_{A}}\right]}^{1/3}},$$

where *M* and $\overline{\upsilon }$ indicate molecular weight (Da) and partial specific volume (mL/g) (subscripts p and d denote protein and detergent, respectively);* ρ* and *η* are the density and viscosity of the aqueous solvent, *δ* is the mass fraction of the bound component (g/g) and *f* is the frictional ratio.

The molecular weight (MW) of C14-betaine detergent was 363.6 Da, the MW of NS4A was 16,452 Da and the specific volume $\overline{\upsilon }$ of NS4A was 0.752 mL/g, as calculated using SEDNTERP^68^. Density and viscosity of the buffer (50 mM Tris, 100 mM NaCl and 33.4% D_2_O) at 25 °C was calculated using SEDNTERP^68^ to be 1.0386 g/mL and 0.9819 cP, respectively. To account for experimental error, we included ± 3% variation in D_2_O concentration (i.e., between 30.4 and 36.4%). The number of bound detergent, the partial specific volume of C14-betaine, and the frictional ratio of the protein was also varied as described in Table 1. The obtained range of s-values is shown in Table 2 (last column).

**Table 1:** Range of values used in variables for the prediction of s-values.

Parameter	Min	Max
C14-betaine *n*_*D*_	0	130^71^
C14-betaine $\overline{\upsilon }$ (mL/g)	0.963*	0.978^65^
Buffer density ρ (g/mL)	1.03532	1.04177
Frictional ratio f/f0**	1.4	1.7

*From our own density matching data.

**See Supplementary Table S1.

**Table 2:** Prediction of s-value range for ZIKV NS4A in C14-betaine, from monomers to hexamers.

	Complex MW (kDa)	Buoyant MW (kDa)	Stokes’ radius (nm)	s-value (s_20,w_) (S)
Monomer	16.5–63.7	2.6–3.6	1.70–2.85	0.55–1.51
Dimer	32.9–80.2	6.1–7.2	2.14–3.04	1.22–2.40
Trimer	49.4–96.6	9.7–10.8	2.45–3.21	1.81–3.15
Tetramer	65.8–113.1	13.2–14.4	2.70–3.36	2.36–3.81
Pentamer	82.3–129.5	16.7–18.0	2.91–3.50	2.87–4.42
Hexamer	98.7–146.0	20.2–21.6	3.09–3.63	3.35–4.99

For sedimentation equilibrium (SE) experiments we used a 4–5 mm solution column. Samples (200 μL) were loaded into AUC cells fitted with a 2-sector epon centerpiece and quartz windows. Multi-speed equilibrium profiles for NS4A were collected at 15,000, 18,000, 22,000, 27,000, and 33,000 rpm. Absorbance data was collected at 280 nm after centrifugation for 18–48 h at each speed. Approach to equilibrium was tested using the Match utility in Heteroanalysis^72^. SE data were fitted to self-equilibrium models in SEDPHAT^73^. A guide to likely molecular weights present in the sample was obtained from a ‘Species Analysis’ test in SEDPHAT by changing the number of species present and floating the molecular weight of these species (see Supplementary Table S2). Data was graphically presented with GUSSI^74^.

### Prediction of NS4A structure

Models of full length NS4A were obtained with ColabFold (AlphaFold2 using MMseqs2)^75^ using the sequence of ZIKV NS4A 1–127, from monomer to higher oligomers. Parameters used were *use_amber* = True, *template_mode* = None, *msa_mode* = MMSeqs2(Uniref + Environmental), *pair_mode* = unpaired + paired, *model_type* = auto, *num_recycles* = 24. For each prediction, best models (rank 1) were selected, according to average pLDDT, and complexes were sorted by pTMscore. Molecule visualization and search of hydrogen bonds and salt bridges were performed with Chimera X^76,77^. Model quality was assessed by two metrics provided by AF2: pLDDT and PAE. The pLDDT (predicted local distance difference test) is a *per-residue* confidence score (> 90 = high confidence, and > 50 = low confidence)^78,79^. Regions with pLDDT > 90 are expected to be modeled with high accuracy, whereas regions with pLDDT < 50 may represent an unstructured region or only structured as part of a complex. The predicted aligned error (PAE) (measured in Ångströms and capped at 31.75 Å) indicates the expected positional error at residue x if the predicted and actual structures are aligned on residue y. Thus, low PAE values (colored generally in blue in a PAE plot) between two domains or subunits represent well-defined relative positions and orientations of these two bodies.

## Results

### Oligomerization of ZIKV NS4A in lipid membranes

**Figure 1:**
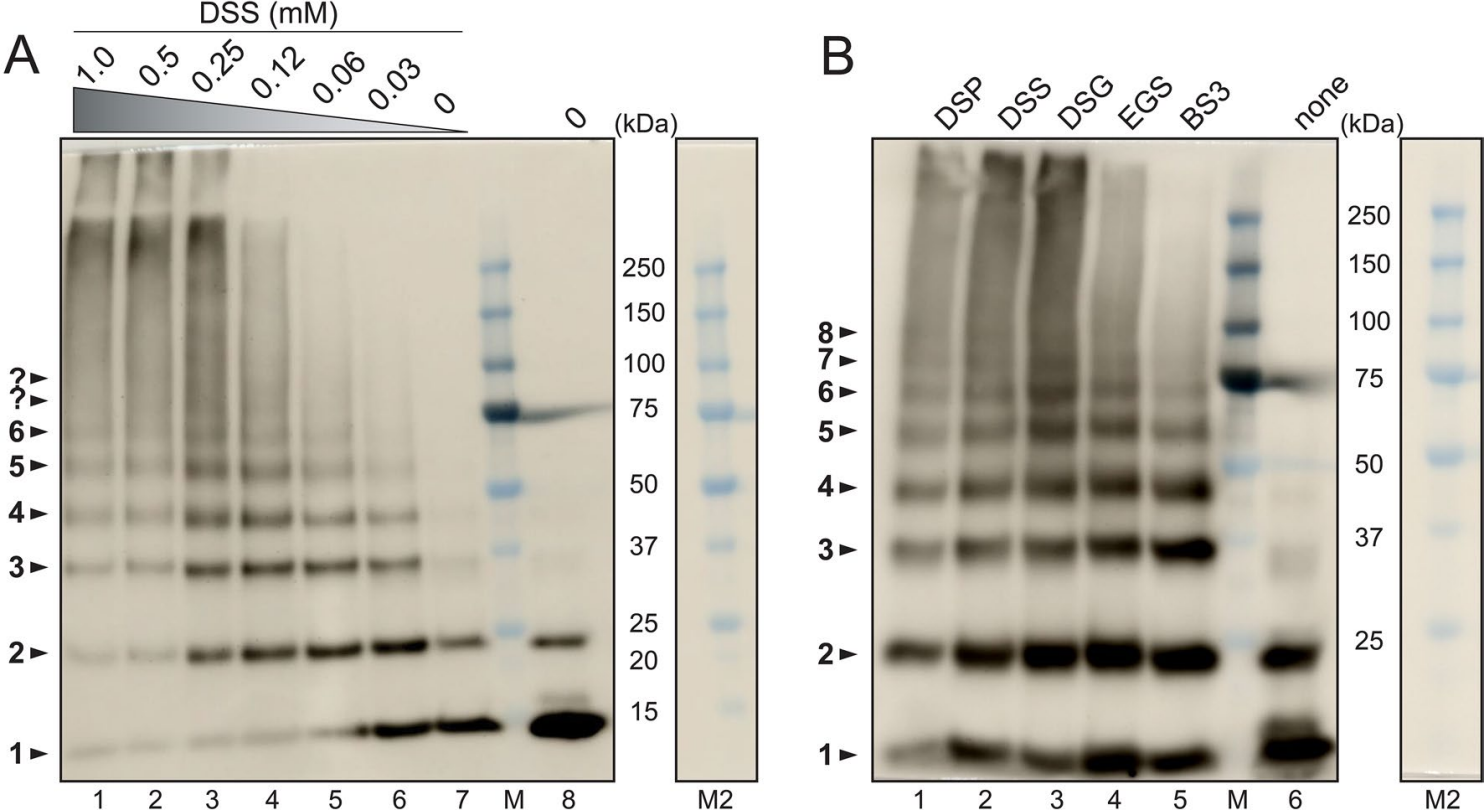
Crosslinking of ZIKV NS4A in lipid membranes. (**A**) Crosslinking of NS4A in PC/PS liposomes with DSS at the concentrations shown (lanes 1–7, see bottom of the gel) and purified non-crosslinked NS4A in DM detergent (lane 8); (**B**) crosslinking of NS4A in PC/PS liposomes with a fixed concentration (0.25 mM) of the crosslinkers shown (lanes 1–5) and purified non-crosslinked NS4A in DM detergent (lane 6). NS4A was detected by Western blot using anti-His-tag antibodies. The gel images are a composite of chemiluminescence and brightfield. Lane M is the MW marker, and lane M2 is lane M without chemiluminescence overlay, shown for clarity.

We suggested previously that both ZIKV NS4A and its N-terminal domain form oligomers larger than dimers in solution^59^, based on size exclusion and dynamic and static light scattering (DLS ad SLS, respectively). However, these techniques do not provide sufficient resolution to confirm the oligomeric size of NS4A. To test this, we reconstituted purified ZIKV NS4A into POPC/POPS liposomes, crosslinked the protein using various crosslinkers, and separated the crosslinked species by SDS-PAGE (Fig. 1).

First, we examined the electrophoretic behaviour of NS4A without crosslinking. In DM detergent, NS4A appeared as a major band consistent with monomers, a minor band predicted to be dimers, and a very faint band of possibly trimers, see Fig. 1A (lane 8) or Fig. 1B (lane 6). When NS4A was reconstituted into liposomes without crosslinking, the intensity of the bands corresponding to oligomers slightly increased, with trimers and tetramers becoming more apparent (Fig. 1A, lane 7). Addition of a membrane-permeable crosslinker DSS at increasing concentrations (Fig. 1A, lanes 1–6) produced a ladder, from monomers to hexamers, even at the lowest DSS concentration (0.03 mM, ie., 2–3 × molar excess to NS4A). With increasing DSS concentration (Fig. 1A, lanes 7 to 1), the monomer band became depleted while higher oligomeric bands became enriched. Oligomer bands larger than hexamers seem to merge into a smear and become more difficult to discern.

We then used various crosslinkers at a fixed 0.25 mM concentration (Fig. 1B, lanes 1–5). All crosslinkers produced oligomer bands up to octamers, except BS3 where the highest observable bands correspond to hexamers and where the smearing pattern seen in other crosslinkers is not observed. To note, BS3 is the only one of these crosslinkers that is membrane-impermeable, and therefore can only access water-accessible residues. This suggests that oligomers higher than hexamers result from crosslinking of membrane-embedded residues. Alternatively, it is possible that oligomers larger than hexamers are artifacts caused by non-specific aggregation. The latter is suggested by the smears observed at high MW, only seen in membrane-permeable crosslinkers. Regardless, these crosslinking results indicate that NS4A forms large oligomers in a lipid environment, at least up to hexamers.

### Sedimentation velocity analysis of ZIKV NS4A

**Figure 2:**
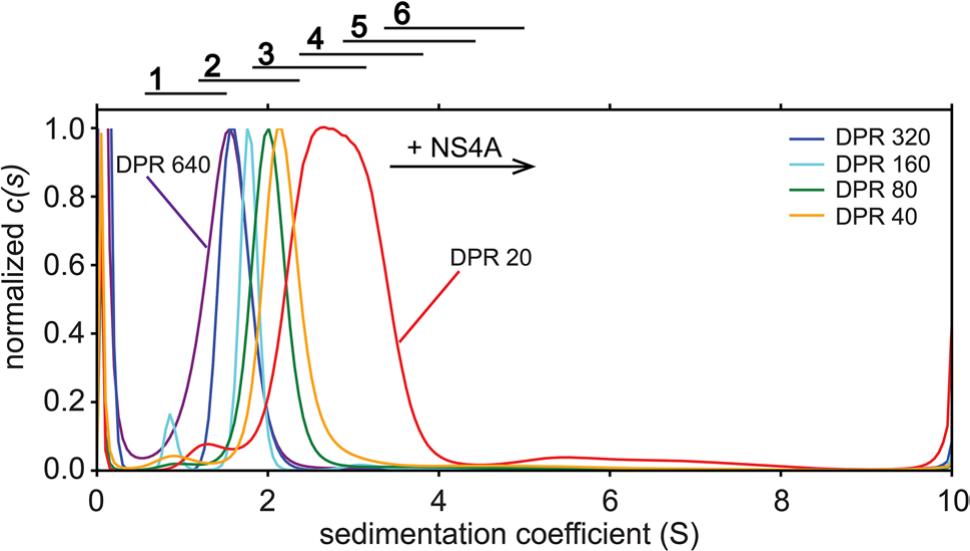
Sedimentation velocity profile of NS4A protein in C14-betaine detergent. Comparison of *c(s)* distributions normalized to main peak height of NS4A at various DPRs (see legend in figure) in the presence of reductant TCEP. Lines above the graph indicate the range of calculated s-values corresponding to each oligomeric size (see “S-value range estimation” in “Methods” section and Table 2).

We next performed sedimentation velocity (SV) experiments on NS4A in a mild detergent C14-betaine (see raw data in Supplementary Fig. S3). In the normalized c(s) plot (Fig. 2), we observed a band (~ 90% of the population) and sometimes a small shoulder, which clearly shifted to a higher s-value with decreasing detergent-to-protein ratio (DPR), i.e., when increasing protein concentration, from approximately 1.5 to 3 S. Single bands with concentration-dependent s-value are typically encountered in conditions of rapid exchange between smaller and larger oligomers, where the s-value observed at each concentration is a weighted average of the individual components^80^. Based on possible variations in the micelle composition, and allowing for experimental error of several parameters (Table 1), we estimated a range of minimum and maximum expected s-values for the different oligomeric types (see “S-value range estimation” in “Methods” section) from monomers to hexamers (Table 2). These values ranges are shown as bars above Fig. 2.

Based on Table 2, the main band in the most diluted samples (DPR 640 and 320) in Fig. 2 likely corresponds to dimers. At the highest protein concentration tested (DPR20), the main band seems to correspond to tetramers or pentamers. However, in a fast-exchange equilibrium the s-value is a weighted average of the components, and the band in the most concentrated sample (DPR 20) does not appear to be the final species, therefore the equilibrium must involve dimers and a species larger than a tetramer/pentamer, possibly a hexamer based on the liposome crosslinking experiments (Fig. 1).

### Sedimentation equilibrium (SE) analysis of ZIKV NS4A

**Figure 3:**
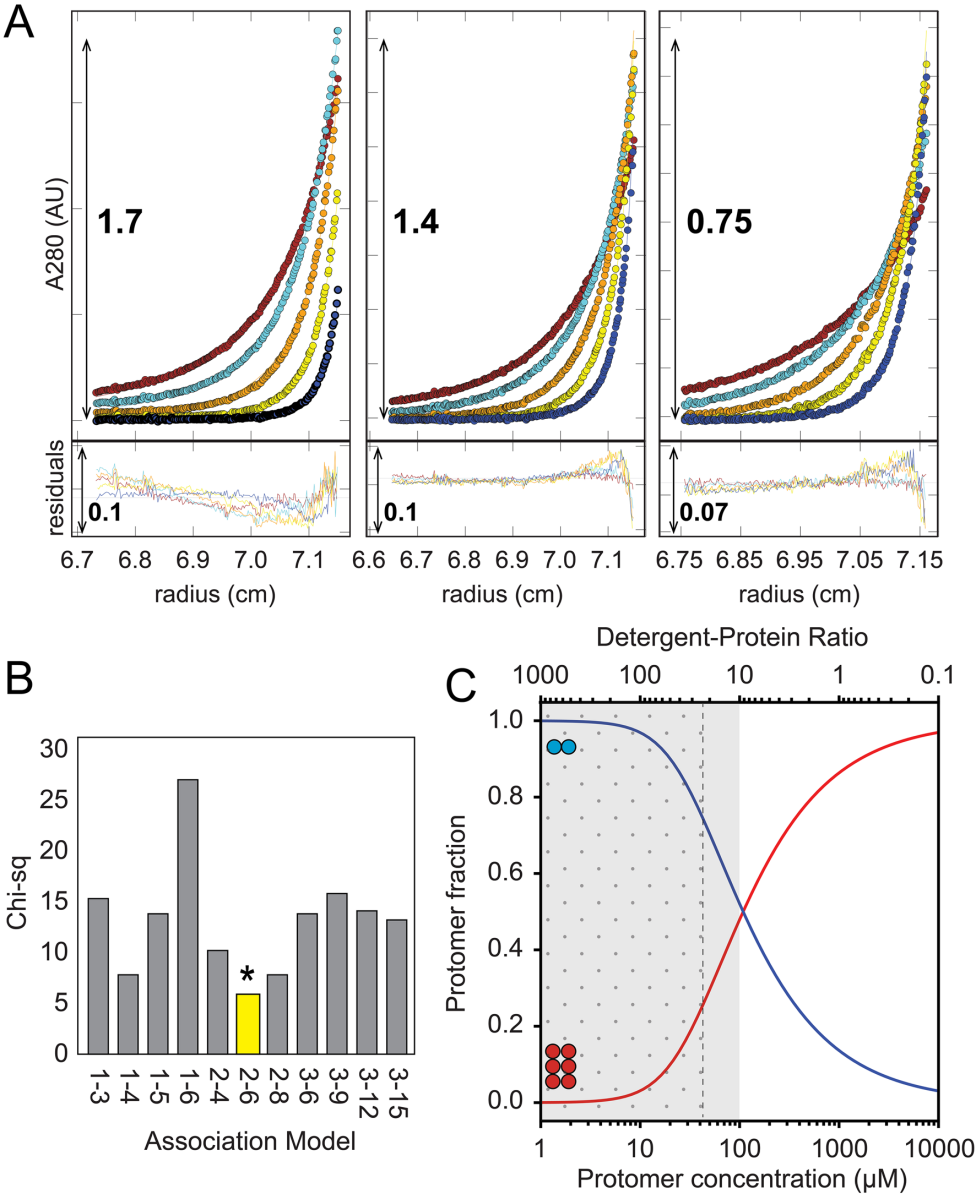
Sedimentation equilibrium of ZIKV NS4A. (**A**) Sedimentation profile (circles) for NS4A loaded from high (left) to low (right) NS4A concentrations (see “Methods” section). The fitted line corresponding to the best fit 2–6 model (see **B**) is hidden below the data points. Fitting residuals in the lower panel show a small amplitude and random distribution; (**B**) Chi-squared values corresponding to several oligomerization models tested (shown in the x-axis). Best fit model (2–6) is shown in yellow. (**C**) Species population plot for the 2–6 model, with the concentration range present in SV and SE experiments indicated by dotted and grey areas, respectively.

The oligomerization behaviour of NS4A was examined further using AUC SE experiments in the presence of reductant TCEP (Fig. 3A). After testing several models (only representative ones are shown here) the data could be best described by a dimer–hexamer equilibrium with log_10_Ka of 8.05, as indicated by the lowest Chi-squared value for this model (Fig. 3B). However, we note that near the bottom of the cell fitting residuals are larger than in the rest of the cell, suggesting that a more complex model may be able to explain the data better. Also, a monomer–dimer–hexamer and a monomer–trimer–hexamer model could fit as well as the dimer-hexamer model, but did not significantly improve the fit (not shown). Lastly, an excellent fit was found with a non-interacting model consisting of a dimer and a pentamer (not shown), but this is inconsistent with SV data which suggests a fast exchange between two oligomeric forms, and with crosslinking results (Fig. 1), where hexamers are clearly observed.

The population plot corresponding to the dimer-hexamer model (Fig. 3C) shows that within the concentration range used and observed in both SV and SE experiments, up to 50% of the dimers participate in the formation of hexamers. A fast exchange between a dimer and a hexamer (which could consist of a trimer of dimers) is consistent with results obtained from both crosslinking (Fig. 1) and SV experiments (Fig. 2).

### Predicted structure of NS4A monomer

**Figure 4:**
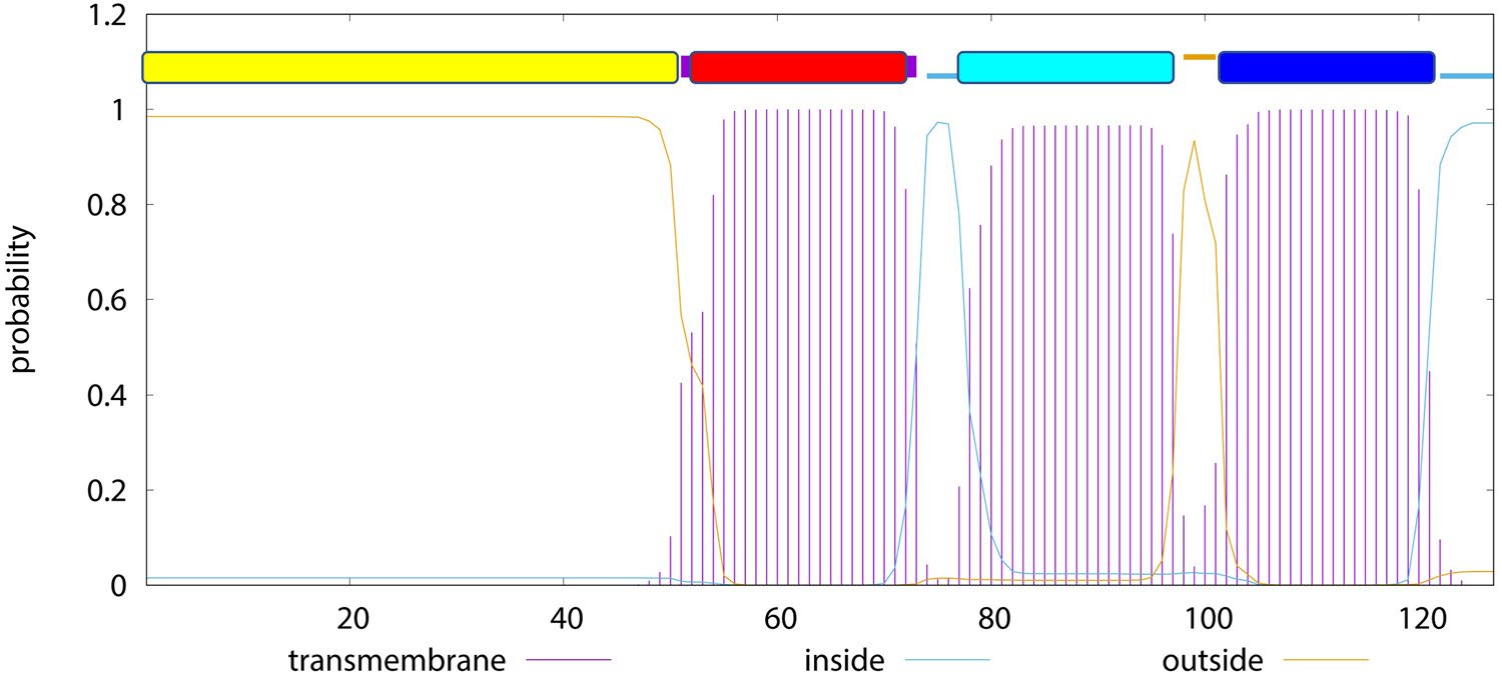
Predicted topology of ZIKV NS4A. Prediction of TM domains and topology using TMHMM. The different parts of the protein are color coded: N-terminal tail (yellow) and the three predicted TM domains in red, cyan, and blue.

The topology of ZIKV NS4A (Fig. 4A) was derived from TMHMM^35^ and the color scheme shown is used in subsequent figures (see below).

**Figure 5:**
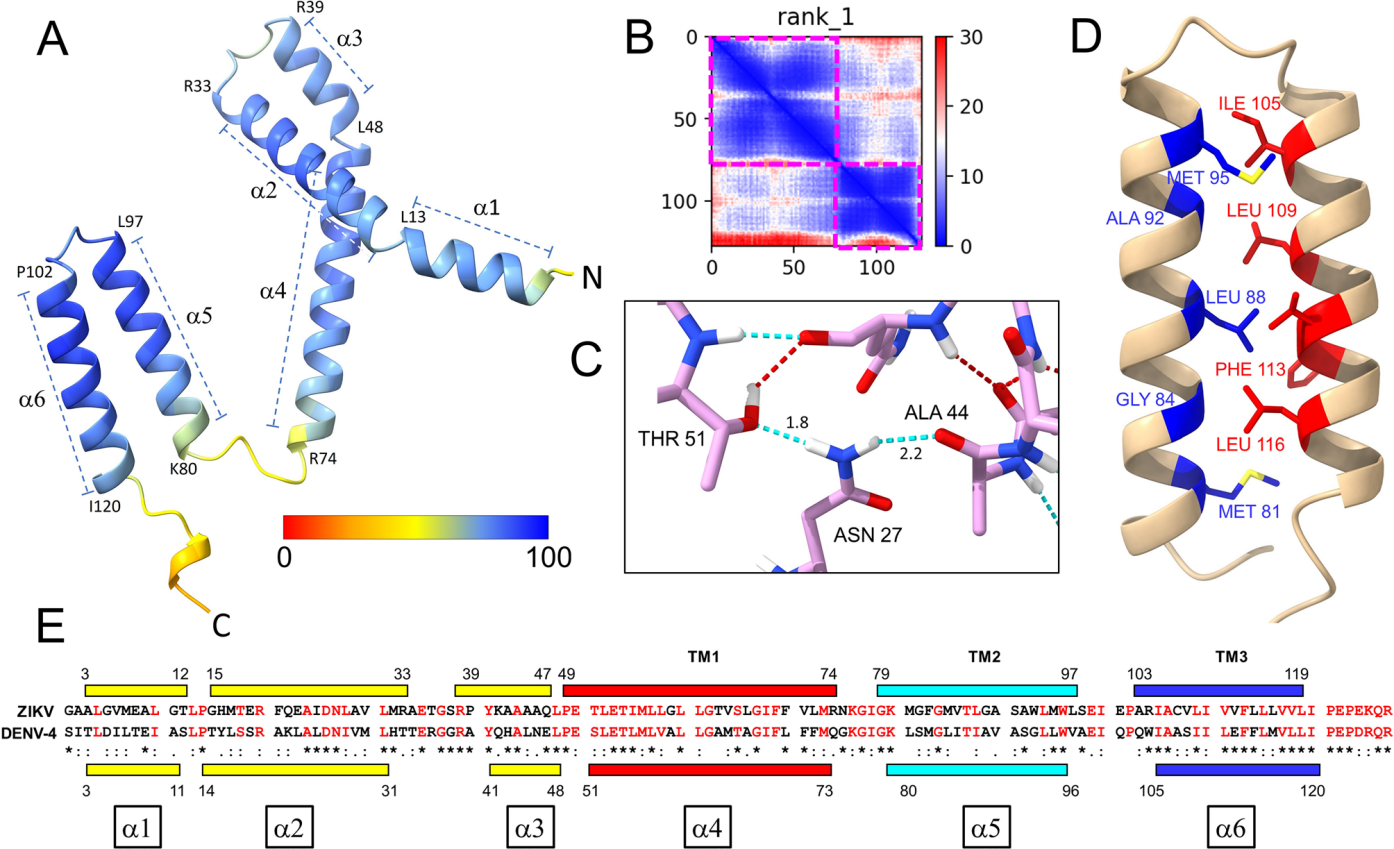
AF-2 structure prediction of monomeric ZIKV NS4A. (**A**) Predicted rank 1 model of NS4A, colored according to Predicted local Distance Difference Test (plDDT) scores (see color bar, where blue is best, red is worst). Most of the residues in the AF2 monomer model showed pLDDT > 90 (see Supplementary Fig. S5); (**B**) PAE plot showing level of confidence in relative positions of residues (low PAE represents high confidence = blue, medium = white, high PAE represents low confidence = red). Confidence within each domain (within the purple squares) is high, but the two domains inside these squares appear disconnected; (**C**) close-up of stabilizing hydrogen bonds in the region α1–α4 obtained using Chimera X, where distance is shown in Å; (**D**) predicted interaction between α5 and α6; (**E**) alignment of the NS4A sequences of ZIKV used here and DEN-4 NS4A. The predicted α-helical domains are shown as bars and are color-coded as in Fig. 4. Those for ZIKV are predicted by AF2, whereas those of DENV-4 were observed experimentally by solution NMR in DPC detergent^45^. Residues identical in both sequences or very similar (T/S or I/L) are shown in red. For reference, the lowest row indicates similarity (., :, *) among the four serotypes of DENV.

Next, we attempted the prediction of the structures of NS4A present in detergent using AlphaFold-2 (AF2)^81,82^. The structure of the NS4A monomer was predicted overall with very high confidence (Fig. 5A, see plDDT color bar and Supplementary Fig. S4). The Predicted Alignment Error (PAE) plot (Fig. 5B) shows that the structure can be divided into two non-interacting domains (surrounded by a violet square): one domain is formed by the regions encompassing the N-terminus and pTM1 (α1–α4), and the other is encompassing pTM2 and pTM3 (α5–α6). The relative position of these two domains is uncertain, as shown by the off-diagonal white colour.

Hydrogen bonds that may stabilize the structure of this monomer in the first domain (Fig. 5C) involve Asn27, Ala44 and Thr51, which therefore must be important for folding and stability of the monomer. Van der Waals interactions at this first domain only involve residues in α2 with α3, or beginning of α4, but not α1. The interactions predicted for the second domain (α5–α6) are shown in Fig. 5D. However, we note that NS4A has been proposed to adopt a ‘U shape’ where α5 (pTM2) does not span the membrane^32^, whereas NMR-based structural data in DPC detergent also showed that the pTM2 region is less protected against H/D exchange^45^. In contrast, this AF2 prediction is more compatible with a regular α-helical bundle with a tight interaction between pTM2 and pTM3 (Fig. 5D). Similar discrepancies between AF2 prediction and experimental data have also been observed in NS2 and NS4B^83^. It is possible that the model shown for the second domain is an artifact, or it may represent an alternative population of NS4A forming an α-helical bundle. Nevertheless, it is remarkable that the position of all α-helical segments is consistent with those reported experimentally from solution NMR^45^ of DENV-4 NS4A (Fig. 5E). AF2 even predicted the presence of the short α-helix 3 (residues 39–47) which was reported as encompassing 41–48 in DENV-4^45^.

### Predicted structure of a NS4A dimer

**Figure 6:**
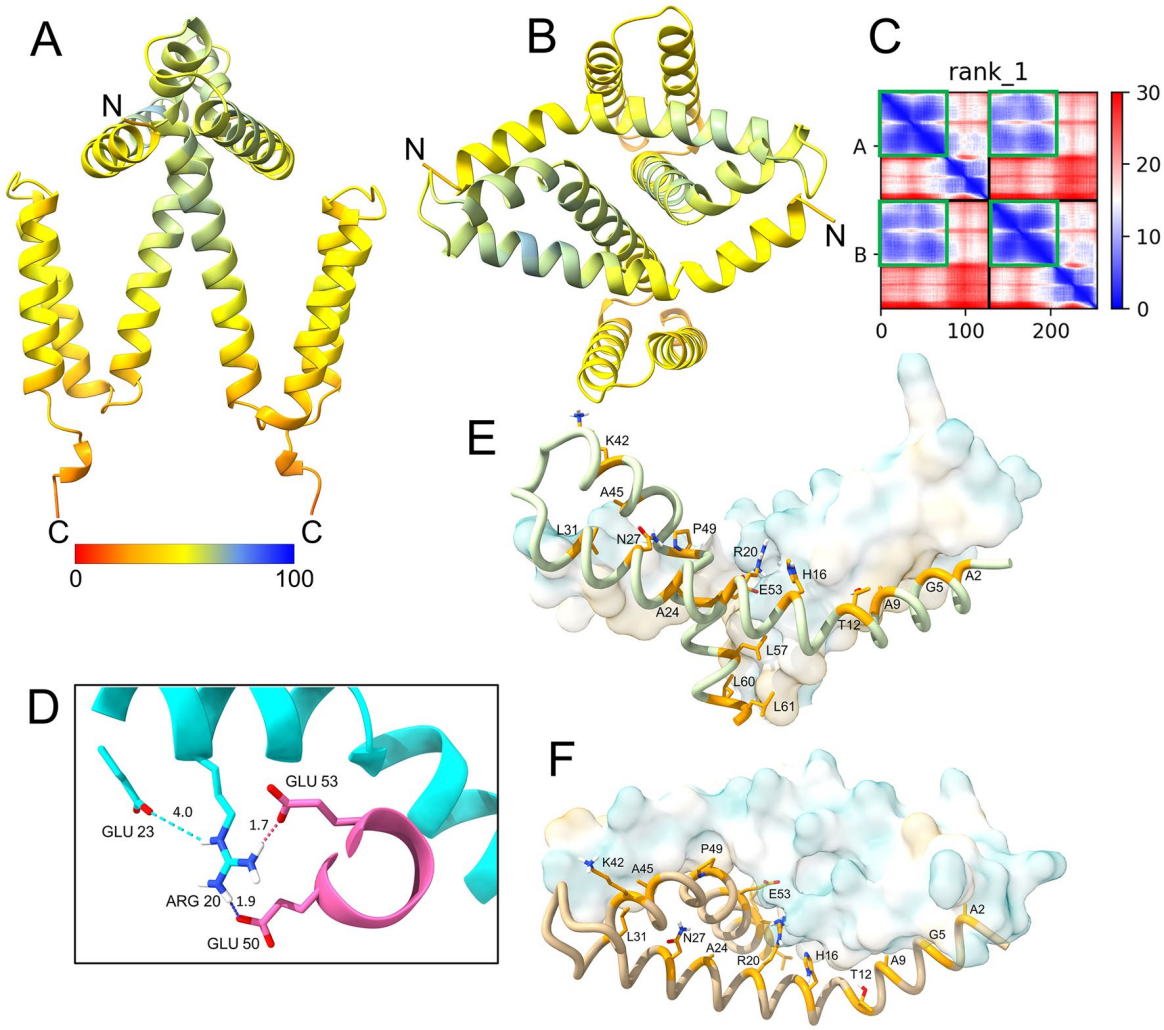
Alphafold-2 structure prediction of dimeric NS4A. (**A**) Side view of predicted rank 1 model of NS4A homodimer colored according to plDDT scores (see color bar). Confidence was relatively high (pLDDT ~ 80) for residues ~ 20–60 (mostly α3 and α4), but low (pLDDT ~ 50) for the rest of the molecule; (**B**) top view of (**A**); (**C**) PAE plot; (**D**) predicted stabilizing salt bridges between E50 and E53 in one monomer (magenta) and R20 in the other (cyan). Distances are shown in Å; (**E,F**) representation of the N-terminal 62 residues involved in the homomeric interaction shown in side-view (**E**) and top-view (**F**). One monomer is shown as hydrophobicity surface (80% transparency) and the other in liquorice representation (preset in Chimera X). The residues facing the other monomer are indicated.

Despite AF2 inability to produce a U shaped model^32^, an NS4A homodimer was also predicted with relatively high confidence in the first domain, which is the domain involved in oligomerization (Fig. 6A,B), with a plDDT of 80 (Supplementary Fig. S4, see n = 2). The PAE plot (Fig. 6C) shows confidence in the relative positions of the first domains (α1–α4) of the two monomers (Fig. 6C, green squares). Salt bridges are predicted to occur between the two monomers, involving Arg20 in one monomer and Glu50 and Glu53 in the other (Fig. 6D). The contacts between the two monomers appear to involve interactions between α1 in one monomer with α3 and α4 in the other monomer (Fig. 6E,F). Residues in α4 in the two monomers also interact (e.g., the interface formed by E53, L57, L60, but not beyond these residues (Fig. 6E). Hence, according to this model, the C-terminal part of α4 (residues 65 to 74) is not involved in homo-dimer formation. Overall, this model is consistent with the experimental observations that both N-terminal tail^36^ and α4, but not α5 or α6, are essential for oligomer formation^48^.

### Predicted structure of NS4A higher oligomers

We tried to obtain models consistent with the higher order oligomers suggested by crosslinking and AUC data, but these did not produce reliable structures (low plDDT, see Supplementary Fig. S4). We hypothesize that this may be caused by the fact that these forms require the formation of a U-shaped model that is not predicted by AF2.

## Discussion

### Experimentally determined oligomeric size

The first identification of a flavivirus NS4A protein was reported in 1989 by Speight and Westaway^84^ and NS4A was suggested to oligomerize as early as 1996, possibly as dimers^47^. Since then, the nature of this NS4A homo-oligomer and the identification of residues involved in monomer–monomer interactions has been debated in the literature.

Our SV experiments conducted using ZIKV NS4A protein in a mild detergent clearly show that the protein forms at least dimers, even in conditions where NS4A is very diluted (DPR ~ 640). A previous NMR study used DENV NS4A in DPC (at DPR 400) and also performed crosslinking in this detergent^45^. In these conditions, clearly most of the protein would have been monomeric or dimeric, explaining why mostly monomers and only some dimers were observed after crosslinking. Our SV data shows that even at DPR 20, 100% conversion to hexamers is not possible in C14-betaine, but hexamers are clearly observed in our crosslinking experiments performed in liposomes at a relatively low lipid-to-protein ratio.

In addition, equilibrium data is fitted optimally with a dimer-hexamer system, and similar results were obtained in DPC detergent (not shown). The presence of hexamers is not inconsistent with the monomers, dimers, trimers or even tetramers previously observed in SDS gels^36,42,46–48^ since SDS is a harsh detergent that would disrupt larger oligomers, or would generate artefactual ones. Nevertheless, these previous works already hinted at the possibility of the presence of NS4A oligomers higher than dimers.

### NS4A oligomerization in the infected cell

It is possible that the trigger for NS4A oligomerization in the infected cell is its separation from NS4B. The complex NS4A/NS4B has been reported to have a Kd of ~ 50 nM^42^ and takes place via residues 40–47 in NS4A (helix α3). Protein NS4A was identified as the key protein responsible for the induction of membrane structures similar to the ones present at latter stages of infection, with a strict requirement for protease NS2B-3pro to cleave NS4A from NS4B at the 4A-2 K site in the NS4A-2K-NS4B polyprotein precursor^44^, possibly aided by a wedge insertion of helix α5 in one leaflet of the membrane^32^. Membrane remodelling is induced at the end of the latent period^46^ and corresponds with the exponential increase in viral RNA. This rearrangement may also require the interaction of the N-terminal domain of NS4A with membranes^38^. However, we note that the N-terminal domain of Zika NS4A, in contrast to that of HCV NS4B^59^ was not able to induce aggregation of liposomes^59^. Unfortunately, AF2 could not find predict NS4A-NS4B interaction, whether separately or with a NS4A-2K-NS4B construct (not shown).

### AF2 predictions of ZIKV NS4A monomer and oligomers

The hydrophilic N-terminal domain of NS4A was initially suggested to be important for homo-oligomerization^36^, but helix α4 (pTM1) was later proposed as the main contributor^48^. Although the latter paper attributed a lower importance to the role of the N-terminal domain, the data was obtained using a pull-down co-immunoprecipitation assay; the small N-terminal domain was fused to a bulky EGFP which may have partially interfered with the interaction. That the N-terminal domain is an important contributor to NS4A oligomerization is also suggested by the ability of this domain to form oligomers in solution^59,85^.

Although in the present work AF2 did not predict a U-shaped conformation for NS4A, it predicts that the N-terminal and C-terminal halves of the protein fold independently, thus the interactions shown in the N-terminal domain (helices α1–α4) may represent true interactions. In this hypothesis, the intermolecular interactions in the dimeric model predicted by AF2 (this paper) only involve the first half of the NS4A sequence This is consistent with previous experimental results, since E50A and G67A, but not R12, P49A and K80A, attenuated replication and reduced oligomerization, whereas residues E53, G66 and G67 were involved in NS4A (peptide 17–80) oligomerization^48^. These results are consistent with the AF2-predicted intermolecular salt bridges in the dimeric model between E50 and R20, and with the high conservation of these two residues (Supplementary Fig. S5); while the first is always acidic, the second is always basic. Also, E53, L60 and G66 are predicted by AF2 to line the side of the α4 helix involved in α4–α4 interactions, whereas no stabilizing hydrogen bonds were found by AF2 for R12, P49A and K80A. According to these AF2 predictions, proper folding of the monomer in the first domain (α1–α4) only involves α2, α3 and the first third of α4 (until residue ~ 60). In the dimer, intermolecular contacts also involve α1, consistent with previous reports^36^ and also α3 and 2/3 of α4 (up to residue 64), also consistent with previous experimental data using DENV-2 NS4A^48^. The N-terminal domain has been mutated extensively and is important for replication and viral production^40,49^, although whether effects are directly related to NS4A oligomerization is not known. Therefore, mutagenesis, biochemical and biophysical data and prediction are consistent with α5 and α6 not participating in NS4A oligomerization. Since AF2 predicts a tight interaction between these two C-terminal helices, which is incompatible with a ‘U’ shaped conformation, the predicted structure of this second domain may be an artefact, explaining the inability of AF2 to predict higher order oligomers (*n* ≥ *3*) with high confidence.

### Summary

Overall, we have studied for the first time the oligomerisation behavior of a flavivirus (ZIKV) NS4A using crosslinking in liposomes, and in a mild detergent that does not disrupt the integrity of higher-order oligomers. Our data conclusively shows that NS4A forms oligomers larger than dimers, at least up to hexamers. Such structure may be observed using cryo-EM methods and may be responsible for membrane remodeling or pore formation. Drugs that destabilize this oligomer could potentially disrupt NS4A-NS4B interactions, since in DENV, NS4B residues 84–146^42^, proposed to interact with residues 40–50 of NS4A, are the target of several drugs in DENV and other flaviviruses^86^.

### Supplementary Information

Supplementary Information.

## Data Availability

The datasets generated during and/or analysed during the current study are available from the corresponding author on reasonable request.
