# Supplementary material for: Flavivirus Zika NS4A protein forms large oligomers in liposomes and in mild detergent
Source: Sci Rep. 2024 May 31;14:12533. doi: 10.1038/s41598-024-63407-y (PMC11143224; doi:10.1038/s41598-024-63407-y)
Supplement: Supplementary file 1 — Supplementary Information. [file 41598_2024_63407_MOESM1_ESM.pdf]

Flavivirus Zika NS4A protein forms large oligomers in liposomes and in mild detergent.

Wahyu Surya, Shwe Sin Honey and Jaume Torres *

Supplementary File.

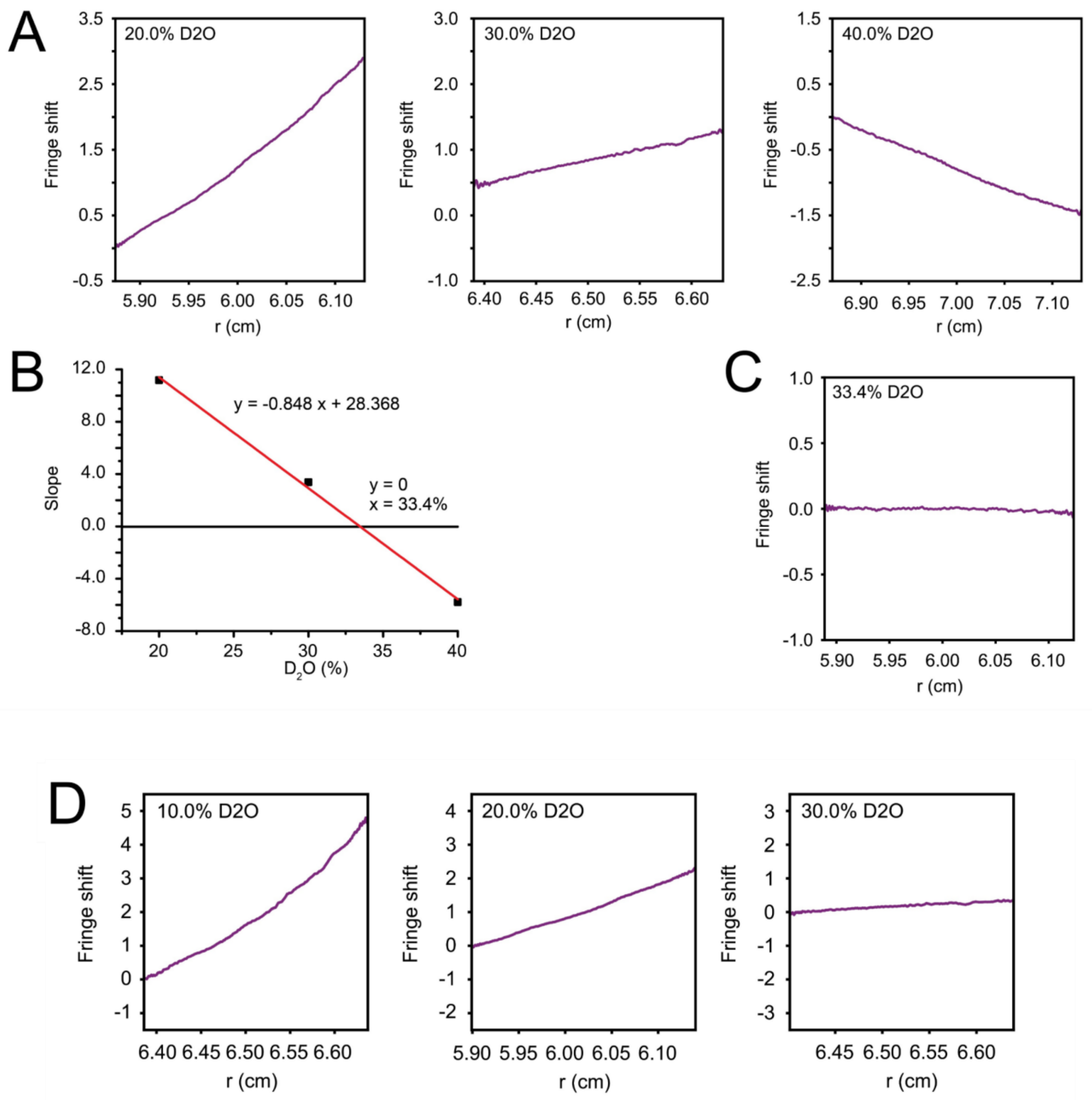

Supplementary Figure S1. (A-C) D_2O density matching experiment resulting in 33.4% D_2O for C14-betaine in 50 mM Tris and 100 mM NaCl; (D) same as A for a buffer with different salt concentrations: 20 mM Tris and 200 mM KCl. Here, the matching D_2O percentage should also be higher than 30% since the detergent density has not been completely matched (fringe shift still has a positive slope).

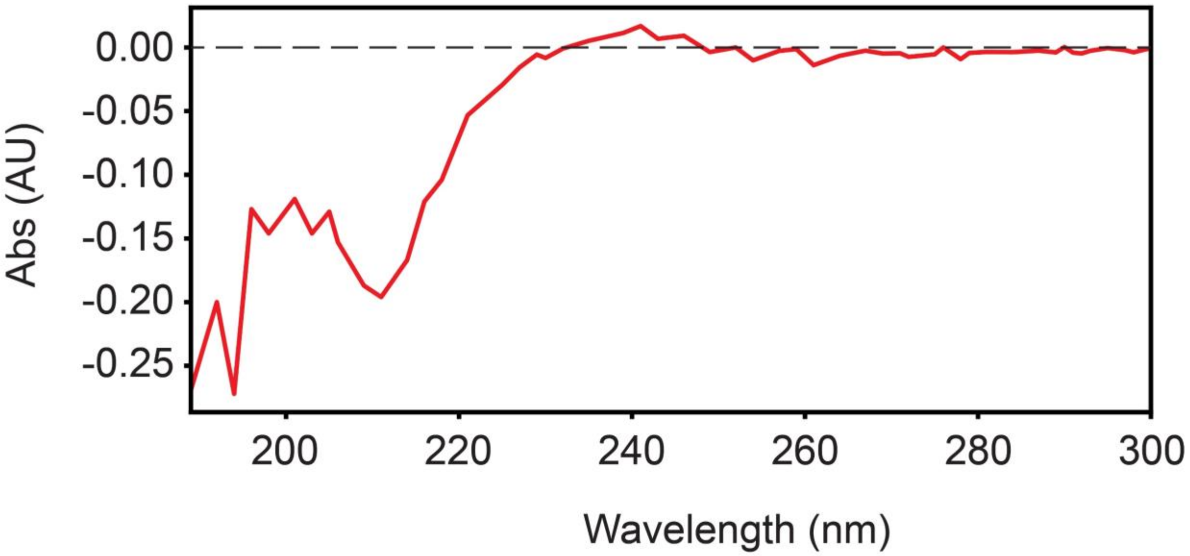

Supplementary Figure S2. UV spectrum of a solution of 5 mM C14-betaine in 50 mM Tris and 100 mM NaCl, collected inside the AUC rotor. The negative values below 220 nm are likely due to either a mismatch in salt concentration between the sample and reference, a lower intensity from the source, or both. Absorbances at 230 and 280 nm are zero.

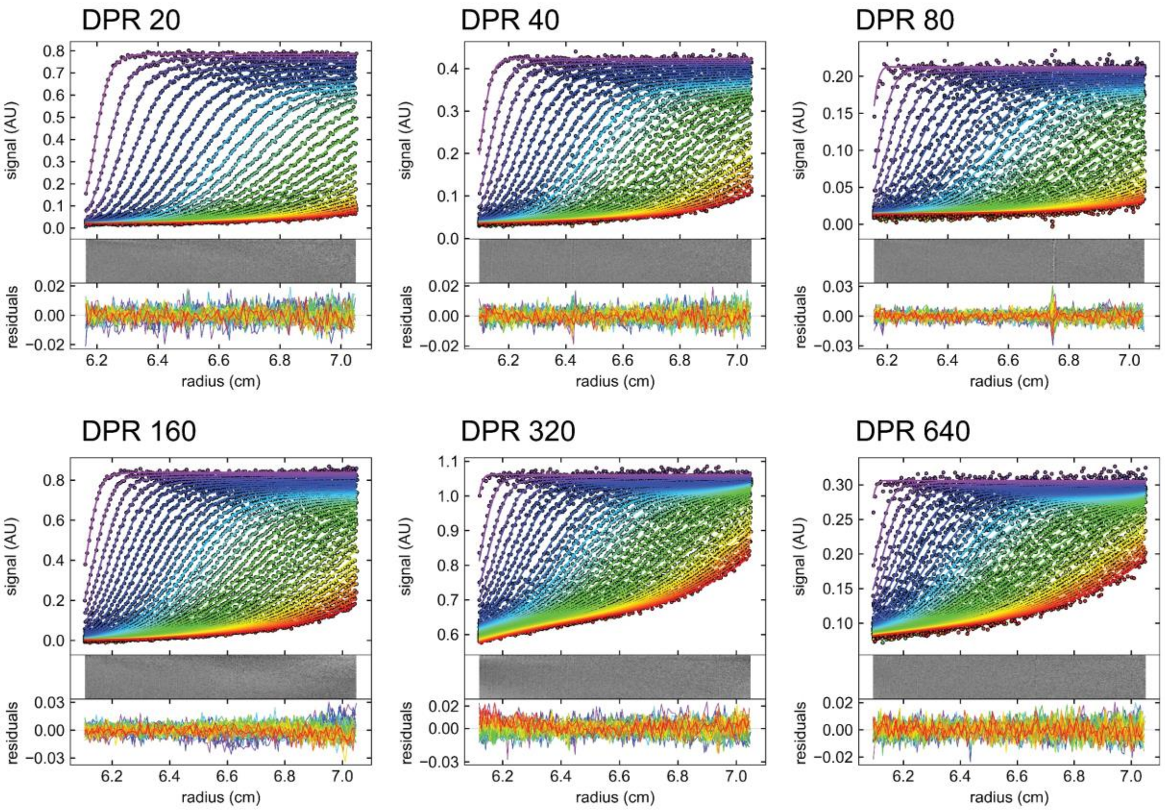

Supplementary Figure S3. Sedimentation velocity raw data for ZIKV NS4A-FL protein. Fitted boundary data (top of each panel) and fitting residuals (2D grayscale in the middle, and 1D in the bottom of each panel) corresponding to NS4A-FL in C14-betaine micelles at the DPR indicated in each panel.

Supplementary Table S1. Frictional ratios. Values of frictional ratio *f/fo* obtained from *c(s)* analysis in SEDFIT.

**DPR**	***f/f0***
640	1.68
320	1.62
160	1.62
80	1.43
40	1.52
20	1.41

Supplementary Table S2. Species analysis results. SE data was examined using the Species Analysis model in SEDPHAT with various number of species and fitting the molecular weight of the species. Models containing more than 1 species were found to fit well, and the simplest model contain only a dimer and a pentamer.

Number of species	Chi-square	Fitted MW (Da)	Oligomeric size (monomer MW = 16452 Da)
**1**	17.5	49,396	3.0
**2**	0.767	32,495<br>75,118	2.0<br>4.6
**3**	0.670	19,823<br>42,201<br>88,637	1.2<br>2.6<br>5.4
**4**	0.651	17,128<br>40,183<br>79,800<br>184,834	1.0<br>2.4<br>4.9<br>11.2

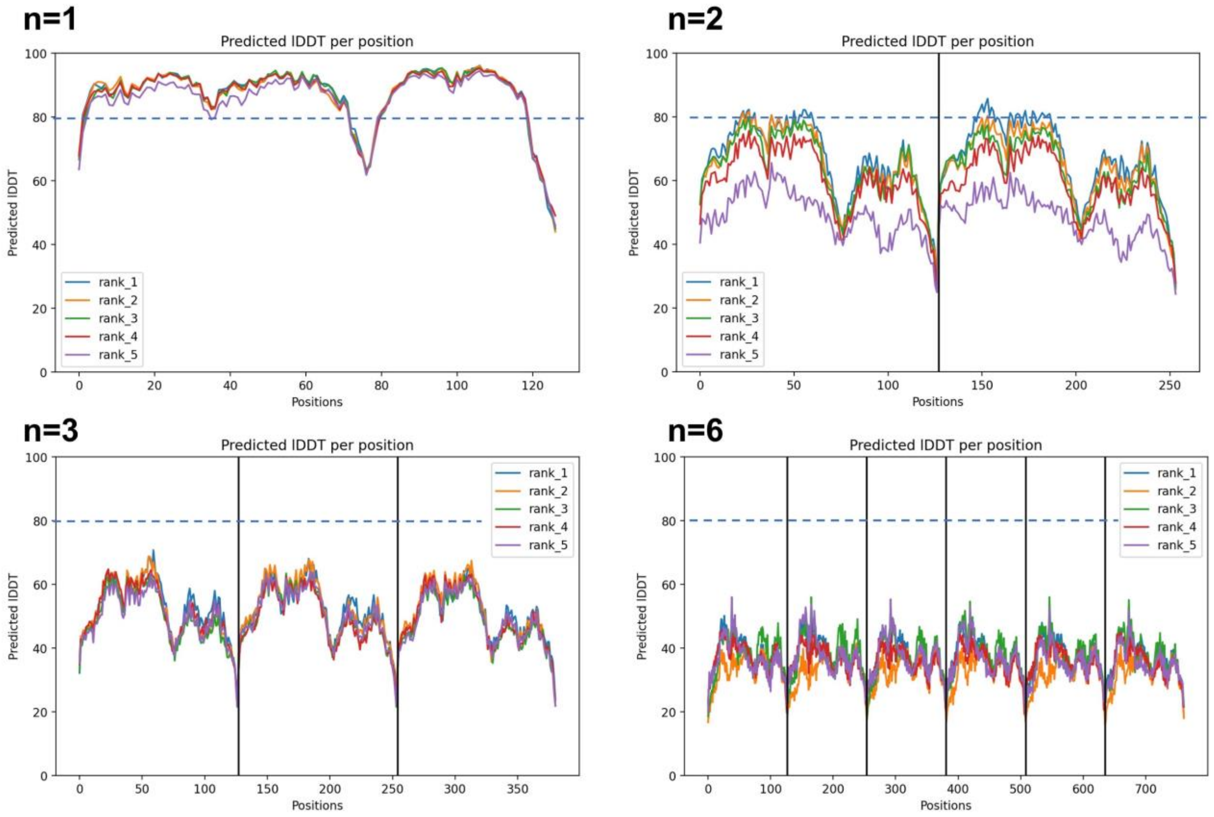

Supplementary Figure S4. Predicted local distance difference test (plDDT) per position for ZIKV NS4A FL monomer and oligomers of increasing size n, as indicated. Scores 100-90 indicate high accuracy comparable to high-resolution structures; regions with scores 70-90 are modelled well with good backbone prediction; regions of scores 50-70 are low confidence and regions <50 cannot be interpreted or may be disordered [1].

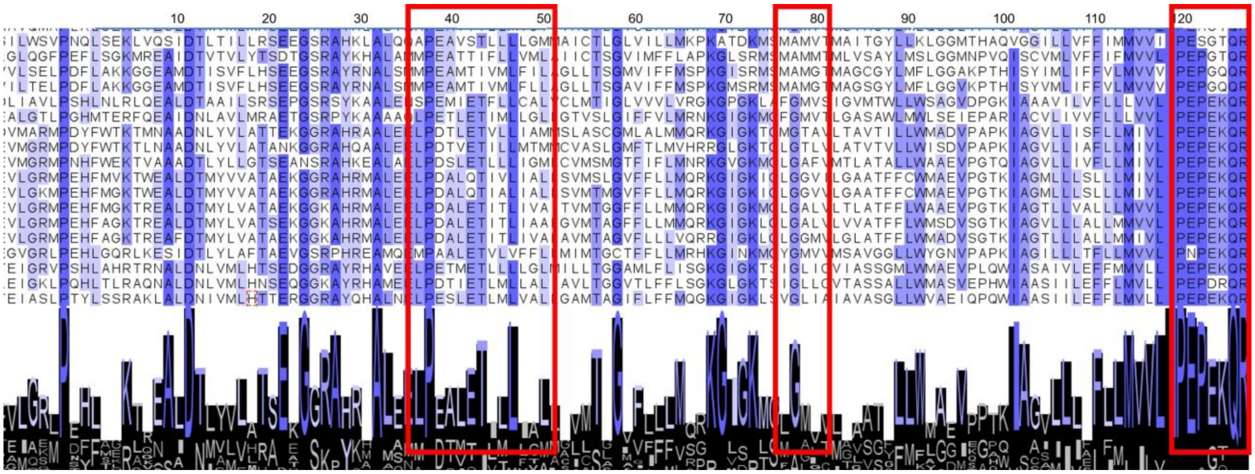

Supplementary Figure S5. Jalview [2] alignment of NS4A in 20 diverse flaviviruses sequences after removing those with >95% redundancy: Zika (ZIKV), Dengue 1-4 (DENV), Yellow Fever (YEFV), Japanese Encephalitis (JAEV), Usutu virus (USUV), Bussuquara virus (BUSV), Rocio virus (ROCV), Banzi (BANV), Edge Hill virus (EHV), Wesselsbron virus (WSLV), Kokobera virus (KOKV), Kunjin virus (KUNV), Ilheus virus (ILHV), West Nile virus (WNV), Saint Louis encephalitis virus (SLEV) and Murray Valley encephalitis virus (MVEV). Columns corresponding to most conserved residues (50% cut-off) are highlighted in blue. Consensus sequence is shown at the bottom, with letter size proportional to conservation. Regions with large clusters of conserved residues are highlighted in red.

1. J. Jumper RE, A. Pritzel, T. Green, M. Figurnov, O. Ronneberger, et al. Highly accurate protein structure prediction with AlphaFold. Nature. 2021;596:583-9. doi: 10.1038/s41586-021-03819-2.
2. Waterhouse AM, Procter JB, Martin DMA, Clamp M, Barton GJ. Jalview Version 2—a multiple sequence alignment editor and analysis workbench. Bioinformatics. 2009;25(9):1189-91. doi: 10.1093/bioinformatics/btp033.
